# Effect of posterior surgery on pre- and post-operative sports activity in patients with adolescent idiopathic scoliosis

**DOI:** 10.1186/1748-7161-10-S1-O31

**Published:** 2015-01-19

**Authors:** Takehide Katogi, Toshiaki Kotani, Jose Miguel T Lumawig, Maiko Sano, Tsutomu Akazawa, Tsuyoshi Sakuma, Shouhei Minami

**Affiliations:** 1 Seirei Sakura Citizen Hospital, Chiba, Japan; 2 Seirei Sakura Citizen Hospital Department of Orthopedic Surgery, Chiba, Japan; 3 St.Luke's Medical Center, Taguig, Philippines

## Background and purpose

Very few papers have been published on the influence of scoliosis surgery on sports activity. The purpose of this study is to analyze the impact of surgery on sports activity in patients with Adolescent Idiopathic Scoliosis (AIS).

## Materials and method

Among the patients with AIS who underwent surgery in our institution, 15 patients were analyzed. Minimum follow up period post-surgery was 2 years. Patients were all females with an average follow-up of 38 months. The mean patient age at surgery was 14.2±1.5 mean Cobb angle of the major curve preoperatively was 54.3±7.4 and 21.8±6.9 degrees postoperatively. We evaluated the strength, speed and agility of these patients pre- and postoperatively using the government-prescribed Physical Fitness Test.

## Results

Postoperatively, general performance in the Physical Fitness Test improved for 4 patients, remained stable for 10 patients and decreased for 1 patient. There were no significant differences seen for the individual physical fitness tests done for these patients pre-and postoperatively (p<0.05).

## Conclusion

Posterior surgery for AIS did not influence the strength, speed and agility of the patients at an average follow up of 38 months post surgery.

